# Assessing performance, calibration, and explainability of machine learning versus traditional models for early outcome prediction after spontaneous intracerebral hemorrhage: a systematic review and meta-analysis protocol

**DOI:** 10.1186/s13643-025-03059-9

**Published:** 2026-01-10

**Authors:** Fan Bu, Rongzhen Xu, Xinyan Zhao, Qiaoxia He, Yandi Wen, Lile Xiong, Lan Qin, Hua Guan

**Affiliations:** 1 Dongzhimen Hospital of Beijing University of Chinese Medicine, Beijing, China; 2 Shenzhen People’s Hospital, Shenzhen, China; 3 Shenzhen Yantian District People’s Hospital, Shenzhen, China

**Keywords:** Intracerebral hemorrhage, Machine learning, Prognostic models, Calibration, Explainability, Systematic review, Meta-analysis

## Abstract

**Background:**

Early outcome prediction after spontaneous intracerebral hemorrhage (ICH) is critical for patient management and counseling. Although machine learning (ML) models are increasingly applied, their comparative performance and explainability relative to traditional statistical models remain unclear.

**Objectives:**

To systematically compare the predictive performance, calibration, and explainability of ML versus traditional models for early outcomes after ICH.

**Methods:**

Following PRISMA-P guidelines and registered in PROSPERO (CRD420251166996), this systematic review and meta-analysis will include studies developing, validating, or comparing ML and traditional models for predicting early mortality or poor functional outcome (mRS ≥ 3 or GOS ≤ 3) after ICH. Data sources will include PubMed, Embase, Scopus, Web of Science, Cochrane CENTRAL, IEEE Xplore, and major Chinese databases (CNKI, Wanfang, VIP, CBM). Two reviewers will independently screen studies, extract data, and assess risk of bias using the PROBAST + AI tool, which extends and replaces the original PROBAST framework for prediction models incorporating machine learning. Pooled analyses will employ random-effects models; confidence in the body of evidence will be summarized using an adapted approach informed by GRADE principles for prognosis evidence.

**Expected results:**

This review will explore whether ML-based models demonstrate differences in discrimination, calibration, and explainability compared with traditional models.

**Conclusions:**

This review will provide a comprehensive, evidence-based assessment of prognostic modeling for ICH, guiding future model design, validation, and clinical application.

**Systematic review registration:**

PROSPERO CRD420251166996

**Supplementary Information:**

The online version contains supplementary material available at 10.1186/s13643-025-03059-9.

## Introduction

Spontaneous intracerebral hemorrhage (ICH) is a severe subtype of stroke characterized by spontaneous rupture of cerebral vessels and bleeding within the brain parenchyma [1]. Despite accounting for only 10–15% of all strokes, ICH contributes disproportionately to stroke-related mortality and long-term disability [2]. Early prediction of poor outcomes, such as death or severe functional dependence, is crucial for informing clinical management, prognostic counseling, and the design of interventional studies.

Over the past decade, machine learning (ML) techniques have gained increasing attention in prognostic modeling for ICH [3, 4]. These methods can handle complex, nonlinear interactions between high-dimensional variables, offering potential advantages over traditional regression-based models [5]. However, evidence regarding their comparative performance, calibration, and explainability remains limited and inconsistent. Existing studies vary substantially in design, sample size, data sources, feature selection, and validation methods, making it difficult to generalize their findings or draw consistent conclusions.

Moreover, recent advances in multimodal data integration—combining clinical, imaging, and laboratory information—have expanded the potential of ML-based prognostic tools. However, the lack of standardized feature definitions, heterogeneous preprocessing pipelines, and inconsistent validation practices across studies has hindered evidence synthesis and clinical translation.

In contrast, traditional statistical models—such as logistic or Cox regression—have been long established in clinical prediction research and remain the reference standard for outcome modeling [6, 7]. Yet, their predictive power may be constrained by linear assumptions and limited capacity to incorporate complex multimodal data. It is therefore essential to systematically evaluate whether ML-based approaches genuinely offer superior predictive value or simply reflect overfitting or data-specific performance gains.

This systematic review and meta-analysis aims to comprehensively evaluate and compare the predictive performance, calibration, and explainability of ML versus traditional models in forecasting early outcomes after spontaneous ICH. By identifying methodological strengths, weaknesses, and reporting gaps, this study will help clarify the clinical utility of ML-based models and guide future research toward robust, interpretable, and clinically meaningful prognostic tools in neurocritical care.

**Fig. 1:**
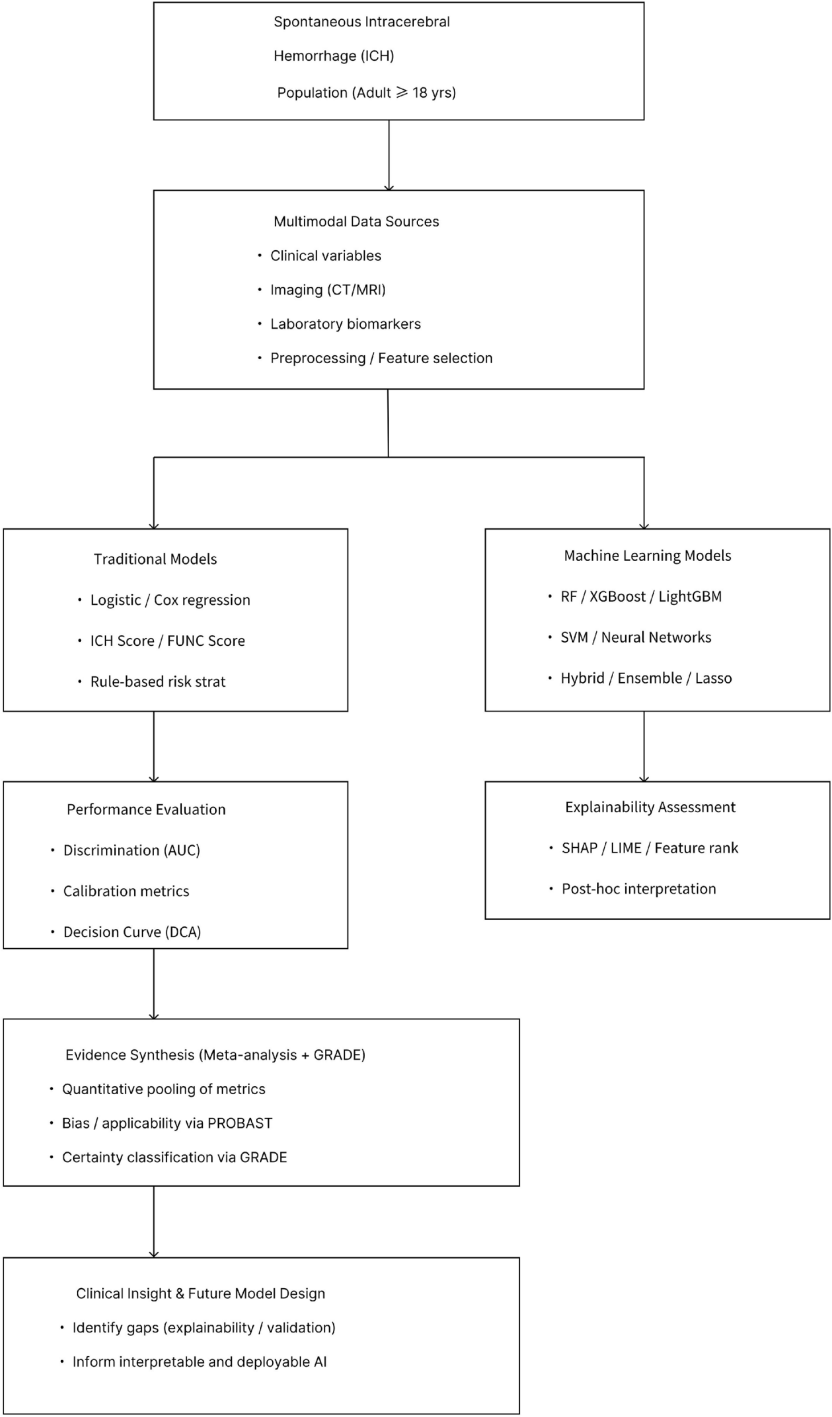
Comparative framework of ML vs. traditional models for ICH outcome prediction

Ultimately, by providing a transparent comparison of ML and traditional models, this review will not only summarize current evidence but also establish a methodological framework to inform future model development, validation, and implementation in clinical neurocritical care. The conceptual framework of this review is shown in Fig. 1, outlining the comparative evaluation between ML and traditional prognostic models.

## Methods

### Protocol and registration

This protocol has been developed in accordance with the PRISMA-P 2015 statement and the Cochrane Handbook for Systematic Reviews of Interventions [8, 9]. The protocol is prospectively registered in PROSPERO (CRD420251166996). All stages of the review—including literature search, study selection, data extraction, risk of bias assessment, and evidence synthesis—will follow predefined procedures to minimize bias and enhance consistency. Any substantive amendments will be justified in the final report and updated in the PROSPERO record.

Because this review synthesizes data from previously published studies, ethical approval is not required. The protocol was internally peer-reviewed by two independent senior researchers in neurocritical care and clinical prediction modeling as part of an institutional, non-commercial funding scheme. This work is supported by existing academic funding and represents a secondary extension of prior research conducted under these grants.

A completed PRISMA-P checklist is provided in Supplementary material S2. Methods for prognosis and prediction model reviews will also be considered where applicable.

### Eligibility criteria

The eligibility criteria were established a priori according to the PICOS framework. Additional detail beyond the PROSPERO record is provided to enhance clarity and reproducibility.

#### Population

Adult patients (≥ 18 years) with spontaneous ICH confirmed by CT or MRI, typically admitted within 24–72 h of symptom onset. No restrictions on sex, ethnicity, country, or healthcare setting.

### Exclusions

Non-spontaneous ICH (traumatic, aneurysmal, vascular malformation–related, or anticoagulant-induced), pediatric populations, animal/preclinical studies, or mixed cohorts without separable ICH data. For studies including mixed adult and pediatric populations, inclusion will require that at least 80% of participants meet the adult eligibility criteria, or that extractable subgroup data for adults are available.

## Intervention (exposure)

Application of ML/AI-based predictive models for early clinical outcomes after ICH, including supervised techniques such as tree-based ensembles (random forest, gradient boosting, XGBoost, LightGBM), SVM, regularized regression (LASSO, ridge, elastic net), neural networks, and hybrid/stacked approaches. Inputs may include clinical, imaging, and laboratory variables [10–12].

Eligible when ML is used for feature selection, multimodal fusion, or model calibration.

### Exclusions

ML used solely for image segmentation or diagnosis without outcome prediction; unsupervised clustering/descriptive analytics only; or purely traditional regression without algorithmic learning/penalization.

#### Comparator (control)

Traditional statistical models or clinical risk scores used for early outcome prediction after ICH (e.g., multivariable logistic regression, Cox models; ICH score, FUNC score, ICH-GS; and other rule-based prognostic tools). Studies must report at least one comparable performance metric (AUC, accuracy, sensitivity, specificity, or calibration indices).

In this review, “traditional models” refer to regression-based or rule-based prognostic models with explicit statistical formulations (e.g., logistic regression, Cox regression, and established clinical risk scores).

### Exclusions

No comparator model; univariable associations only; or non-outcome tasks (e.g., hematoma expansion prediction alone).

#### Outcomes

Primary evaluation dimensions:

- Predictive performance (discrimination).
- AUC, accuracy, sensitivity, specificity, C-statistic.
- Calibration
- Brier score, calibration slope, intercept.
- Explainability
- variable importance and post hoc methods (e.g., SHAP, LIME) [13].
- Early clinical outcomes
- Mortality
- all-cause death within 30–90 days after ICH;
- Functional outcome
- mRS or GOS, with poor outcome defined as mRS ≥ 3 or GOS ≤ 3.

#### Secondary outcomes

Sample size and outcome prevalence; data source (single- vs multicenter); study design (retrospective vs prospective); feature selection and preprocessing; validation type (internal vs external); handling of missing data and class imbalance.

Where available, we will extract decision-analytic measures—including net benefit and decision-curve analysis (DCA)—to contextualize model performance at clinically relevant threshold probabilities. If DCA is not reported, we will compute it when sufficient aggregate data are provided.

### Study design and setting

#### Eligible designs

Original, non-randomized studies developing, validating, or comparing predictive models (retrospective/prospective cohorts; registry-based/multicenter observational studies; secondary analyses of RCT datasets or large databases).

#### Exclusions

Reviews, meta-analyses, editorials, commentaries, conference abstracts without sufficient methodological detail, case reports, animal studies. Clinical/hospital settings only; no geographic or income-level restrictions.

To ensure methodological robustness, studies will be required to include at least 10 outcome events for model development or validation.

### Information sources and search strategy

A comprehensive search will follow the Cochrane Handbook and PRISMA-P 2015 standards.

#### Electronic databases

From inception to final search date, we will search.

PubMed/MEDLINE; Embase (Embase.com); Cochrane CENTRAL; Scopus; Web of Science – SCI; IEEE Xplore; CNKI; Wanfang; VIP (CQVIP); CBM.

No restrictions on language, year, or country. Inclusion of Chinese databases mitigates language/publication bias and improves global representativeness [14].

#### Search strategy

Controlled vocabulary (MeSH/Emtree) and free-text terms related to ICH, ML/AI, and outcome prediction will be used with database-specific syntax.

The complete search strings for all databases are provided in Supplementary material S1.

The search will undergo independent PRESS peer review and will be re-run within two weeks prior to data extraction to capture newly indexed studies; any incremental inclusions will be tracked and reported in an updated PRISMA flow.

#### Grey literature and additional sources

Searches will include ProQuest Dissertations and Theses Global, preprint servers (medRxiv, arXiv), conference proceedings (neurology/stroke/AI), and backward citation tracking. Citation alerts will be set in PubMed and Scopus.

For non-English/Chinese reports, titles/abstracts will be screened using machine translation; potentially eligible full texts will be translated by bilingual researchers or professional services. Translation decisions and uncertainties will be logged and presented in Supplementary material.

#### Study selection process

Records will be de-duplicated in EndNote and screened in Rayyan by two independent reviewers (titles/abstracts, then full texts). Disagreements will be resolved by consensus or a third reviewer.

**Fig. 2:**
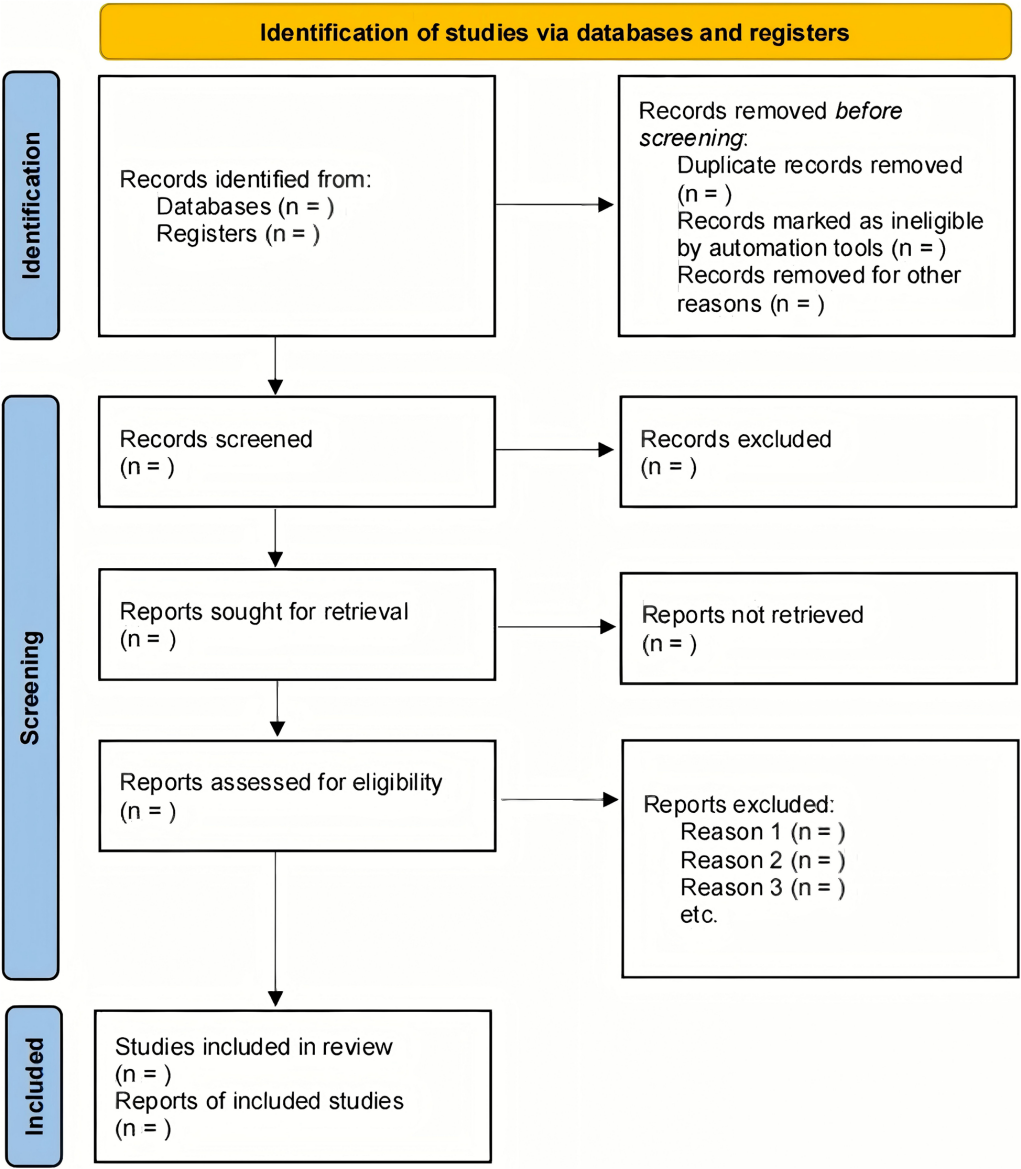
PRISMA 2020 flow diagram illustrating the literature screening and study selection process

The study selection process will be illustrated in a PRISMA 2020 flow diagram (Fig. 2) documenting the number of records identified, screened, excluded, and included at each stage.

#### Quality assurance of the search

The strategy will undergo PRESS 2015 peer review. All modifications will be documented, and the search will be rerun immediately before data extraction.

### Study selection and data management

#### Study selection

Two reviewers will independently screen records against eligibility criteria; full texts will be assessed for inclusion. Inter-reviewer agreement will be quantified using Cohen’s κ (κ ≥ 0.80 as excellent).

#### Data extraction

A piloted Excel form will capture.

Study details; participant characteristics; model characteristics; performance metrics; explainability methods; and study-level covariates. Multiple models per study will be extracted separately. Authors will be contacted for clarifications where needed. Cross-checks will resolve discrepancies.

To avoid double counting, we will identify overlapping cohorts (by institution, timeframe, registry) and preferentially include the most comprehensive or externally validated report. When multiple models per study are reported, we will pre-specify a hierarchy—externally validated > internally validated > apparent performance—and extract all models for qualitative synthesis while nominating one “index model” per study for primary pooling.

We will extract and document how missing data were handled in each included study. No imputation will be performed at the review level; all analyses will rely on reported study-level results.

#### Data management and storage

EndNote will manage references; Rayyan for screening; Excel for extraction/QA. A master decisions log will ensure auditability. Data will be stored on secure institutional servers with restricted access and version control. Upon review completion, the finalized dataset will be archived and may be made available to qualified researchers upon reasonable request following publication.

### Risk of bias and quality assessment

The methodological quality and risk of bias of the included studies will be assessed using PROBAST + AI [15], which is specifically designed for evaluating the development and validation of prediction models. PROBAST + AI formally extends and replaces PROBAST-2019 by addressing additional sources of bias specific to machine learning and artificial intelligence–based prediction models. The use of PROBAST + AI ensures a structured and transparent evaluation of risk of bias and applicability across both ML and traditional statistical prediction models.

In parallel with PROBAST, we will pilot a brief TRIPOD-AI/PROBAST-AI–informed checklist focusing on AI-specific reporting domains, including predictor handling, feature leakage safeguards, preprocessing transparency, hyperparameter tuning, and external validation. This checklist will be used to qualitatively summarize the sufficiency of AI-related reporting; it will not alter PROBAST domain ratings but will be presented descriptively to highlight reporting consistency and completeness.

#### Assessment domains

PROBAST + AI evaluates key domains that contribute to potential bias and applicability concerns.

Participants, Predictors, Outcome, and Analysis [16]. Each signaling question within these domains will be answered as “Yes,” “Probably Yes,” “Probably No,” “No,” or “No information,” leading to a final judgment of low, high, or unclear risk of bias for each domain and overall.

#### Assessment process

Two reviewers will independently assess each included study using a standardized PROBAST + AI checklist, following a calibration exercise on a subset of studies. Disagreements will be resolved by discussion or consultation with a third reviewer with expertise in clinical prediction modeling. Development and validation phases will be evaluated separately when both are reported. Inter-rater reliability will be quantified using Cohen’s κ (κ ≥ 0.80 = excellent).

#### Presentation of quality assessment

Results will be presented in summary tables and as visualization plots generated in R 4.3 using the robvis package. Where feasible, sensitivity analyses will be conducted excluding studies at high risk of bias to examine the robustness of pooled estimates.

### Data synthesis and statistical analysis

#### Overview

We will perform structured synthesis, quantitative meta-analysis where appropriate, and otherwise narrative synthesis (Cochrane guidance).

Analyses: R 4.3, packages metafor and meta.

#### Quantitative synthesis

With ≥ 3 comparable studies (e.g., AUC, C-statistic, calibration slope/Brier), random-effects (DerSimonian–Laird) pooling will be used; REML as a robustness check [7]. AUC may be logit-transformed; proportions via Freeman–Tukey transformation. Report 95% CIs.

When studies report multiple test sets from the same cohort, we will avoid dependency by selecting a single non-overlapping test set per cohort. For AUC pooling, if necessary, we will harmonize time horizons (e.g., 30–90 days) using author-reported aligned estimates; unmatched horizons will be summarized narratively.

#### Heterogeneity

Cochran’s *Q *and *I*^2^ (25/50/75% = low/moderate/high).

If *I*^2^ > 75%, explore via subgroup/meta-regression [17]. Prespecified subgroups: model type; data source; validation type; region; outcome type.

Planned moderators for meta-regression include:

Sample size (log-transformed), outcome prevalence, external vs internal validation, imaging inclusion (yes/no), multimodal inputs (clinical + imaging + labs), and region (Asia vs non-Asia). Meta-regression will be performed only when ≥ 10 studies contribute to a moderator.

#### Sensitivity analyses

Exclude high risk-of-bias studies; exclude small samples/lacking external validation [18]; apply alternative estimators (e.g., Hartung–Knapp–Sidik–Jonkman). Compare with main analyses.

We will construct a bias-resistant analysis set excluding studies at high risk of bias (overall PROBAST + AI“high”), studies without any form of validation, and studies with extreme class imbalance (outcome prevalence < 10% or > 90%) unless appropriate rebalancing was applied and reported.

#### Narrative synthesis

If pooling is infeasible, provide a structured narrative across performance, calibration, and explainability, with tables/figures.

#### Reporting bias and small-study effects

Formal funnel plots and statistical tests for funnel plot asymmetry were developed primarily for intervention-effect meta-analyses and are not appropriate for prediction model performance syntheses. Therefore, they will not be applied in this review. Instead, potential reporting bias will be assessed qualitatively by examining study characteristics, completeness of reporting, and availability of protocols or registrations.

#### Statistical significance

Two-sided tests; α = 0.05. Report per PRISMA 2020, with full code/parameters for reproducibility.

### Confidence in the body of evidence

There is no single official GRADE approach specifically for prediction model performance syntheses. Accordingly, the following framework is intended to provide a descriptive and supportive summary, rather than a formal GRADE rating. We will therefore summarize confidence in the body of evidence using an adapted framework informed by GRADE principles for prognosis evidence, considering risk of bias (PROBAST + AI), inconsistency, indirectness, imprecision, and potential reporting bias [19]. Evidence summaries will be descriptively categorized (e.g., higher vs lower confidence) to aid interpretation, without implying formal GRADE ratings. Two reviewers will independently assess and summarize confidence in the body of evidence with calibration and consensus resolution. Summary tables will be prepared using structured evidence profiles; software such as GRADEpro GDT may be used solely as a reporting aid, without assigning formal GRADE certainty ratings [20]. 

We will adapt GRADE wording to the prediction context.

Inconsistency will follow *I*^2^ and overlap of confidence intervals; indirectness will consider deviation from spontaneous ICH populations or early time windows; imprecision will incorporate optimal information size for AUC differences ≥ 0.05; we will note situations where findings appear consistent across externally validated studies and clinically meaningful, while avoiding formal upgrading/downgrading decisions.

### Patient and public involvement

No patients or members of the public were directly involved in the design, conduct, or reporting of this protocol. To enhance clinical relevance, outcome selection and interpretation frameworks were informed by consultation with clinical neurologists and data scientists experienced in ICH management and prognostic modeling.

Upon publication, we aim to deposit the cleaned extraction sheet, analytic code, and figure scripts in an open repository (e.g., OSF/GitHub) with a permanent DOI; any licensed database search strings will be shared in full in Supplementary material to maximize reproducibility.

## Expected results

This systematic review and meta-analysis is expected to provide a comprehensive synthesis of the current evidence comparing ML and traditional statistical models for early outcome prediction following spontaneous ICH.

This review will summarize and compare reported discrimination and calibration of ML-based and traditional models, and explore whether performance profiles differ across model types and validation settings (e.g., internal vs. external validation and multimodal vs. unimodal inputs) [21].

Furthermore, the review is expected to highlight important heterogeneity in model design and validation practices across studies.

Specifically, differences are likely to emerge in sample size, feature selection techniques, data preprocessing strategies, and external validation efforts [22].

These methodological variations are anticipated to influence both predictive performance and generalizability of ML models.

The review will also summarize the degree to which model explainability has been incorporated into current ML approaches.

We will describe the extent to which interpretability techniques (e.g., SHAP, LIME, feature importance) are reported and evaluated. We will also identify gaps in explainability reporting and assessment across studies [23].

By quantitatively pooling available performance metrics, the meta-analysis will generate pooled estimates of discrimination and calibration, offering an evidence-based benchmark for future prognostic modeling efforts in ICH.

Subgroup and sensitivity analyses are expected to identify factors—such as validation type, model complexity, and study quality—that account for observed heterogeneity and may guide the design of more robust prediction frameworks.

Ultimately, this review aims to produce an evidence-informed summary of the strengths, limitations, and clinical applicability of ML-based versus traditional models.

The results are expected to inform clinical researchers, neurologists, and data scientists about the practical potential and current methodological gaps in applying ML for early prognostication after ICH, providing direction for future studies and the development of clinically implementable predictive tools.

## Discussion and significance

### Rationale and expected contribution

Spontaneous ICH remains one of the most devastating subtypes of stroke, characterized by high mortality and long-term disability despite advances in acute care. Accurate early prognostication is therefore crucial for guiding treatment decisions, resource allocation, and family counseling [24].

In recent years, the rapid growth of ML and artificial intelligence (AI) methods has offered new opportunities for enhancing prognostic modeling in neurological disorders. However, their comparative performance against traditional statistical models—and their interpretability in clinical contexts—remains incompletely understood.

This review will systematically evaluate and synthesize the available evidence on ML and traditional models for early outcome prediction after ICH. By quantitatively comparing predictive performance, calibration, and explainability, the study aims to clarify whether ML approaches truly offer superior predictive value or complementary advantages in clinical decision-making [25, 26]. The findings are expected to provide a methodologically rigorous and evidence-based overview that bridges the gap between computational innovation and bedside applicability.

### Innovation and significance

The proposed review has several innovative aspects:

#### Comprehensive methodological scope

It integrates both English- and Chinese-language databases, thereby minimizing publication and language bias and ensuring global representativeness of the included evidence.

#### Tri-dimensional evaluation framework

It concurrently examines performance, calibration, and explainability, reflecting a holistic view of model quality rather than focusing solely on discrimination metrics.

#### Standardized quality appraisal

Risk of bias will be assessed using PROBAST + AI, and confidence in the body of evidence will be summarized using an adapted, GRADE-informed descriptive approach (without formal GRADE ratings).

#### Translational potential

By identifying methodological strengths and weaknesses, the findings will inform the development of future ML models that are both accurate and clinically interpretable, facilitating responsible implementation in neurocritical care [27].

Together, these features will make this review one of the first comprehensive efforts to systematically appraise ML versus traditional models for ICH outcome prediction.

### Limitations and anticipated challenges

Several challenges are anticipated.

First, heterogeneity across studies in terms of patient populations, imaging protocols, model development procedures, and outcome definitions may limit the feasibility of quantitative pooling [28].

Second, reporting quality and incomplete data (e.g., missing calibration metrics or external validation) may hinder direct comparison between models.

Third, the rapid evolution of ML methods may lead to time-related publication bias, as newer algorithms may not yet be extensively validated or published.

To mitigate these challenges, the review will apply robust inclusion criteria, standardized data extraction templates, and sensitivity analyses to ensure methodological consistency and interpretative clarity.

Where quantitative synthesis is not feasible, a structured narrative synthesis will be presented to summarize patterns and highlight key research gaps.

### Future directions and clinical relevance

The findings of this review are expected to guide both researchers and clinicians toward more reliable and interpretable predictive modeling practices [29].

Future research should focus on:

Conducting prospective, multicenter validations of ML-based prognostic tools [30];

Integrating multimodal data (e.g., clinical, imaging, and biomarker information) to enhance model robustness [31]; and.

Establishing standardized reporting frameworks (e.g., TRIPOD-AI, PROBAST-AI) to improve transparency and reproducibility in prognostic modeling [32, 33].

Ultimately, this review will contribute to advancing precision medicine in neurocritical care, promoting the responsible use of ML in the management of ICH, and supporting the translation of data-driven models into clinical practice.

## Supplementary Information

Supplementary Material 1. Search Strategy. Detailed database search strategies including MeSH/Emtree terms and Boolean operators.

Supplementary Material 2. PRISMA-P 2015 Checklist. Completed checklist indicating adherence to each PRISMA-P item.

## Data Availability

No new data were generated or analyzed in this study. Data supporting the findings of this protocol will be obtained from previously published studies. Upon completion of the review, the finalized extraction dataset and analysis files will be available from the corresponding author upon reasonable request.

## Abbreviations

AI: Artificial intelligence
AUC: Area under the curve
CBM: Chinese Biomedical Literature Database
CENTRAL: Cochrane Central Register of Controlled Trials
CNKI: China National Knowledge Infrastructure
DCA: Decision Curve Analysis
GOS: Glasgow Outcome Scale
GRADE: Grading of Recommendations, Assessment, Development, and Evaluation
ICH: Intracerebral hemorrhage
IEEE: Institute of Electrical and Electronics Engineers
I^2^: I-squared statistic (measure of heterogeneity)
LASSO: Least Absolute Shrinkage and Selection Operator
LightGBM: Light Gradient Boosting Machine
ML: Machine learning
mRS: Modified Rankin Scale
OR: Odds ratio
PICOS: Population, Intervention, Comparator, Outcomes, and Study design
PRISMA: Preferred Reporting Items for Systematic Reviews and Meta-Analyses
PRISMA-P: Preferred Reporting Items for Systematic Review and Meta-Analysis Protocols
PROBAST: Prediction Model Risk of Bias Assessment Tool
PROSPERO: International Prospective Register of Systematic Reviews
RF: Random Forest
RCT: Randomized controlled trial
REML: Restricted maximum likelihood
SVM: Support vector machine
TRIPOD: Transparent Reporting of a multivariable prediction model for Individual Prognosis or Diagnosis
TRIPOD-AI: TRIPOD extension for Artificial Intelligence-based models
VIP: Chongqing VIP Information Database
Web of Science–SCI: Science Citation Index Expanded
95% CI: 95% Confidence interval

## References

[1] GBD 2019 Stroke Collaborators. Global, regional, and national burden of stroke and its risk factors, 1990–2019: a systematic analysis for the Global Burden of Disease Study 2019. Lancet Neurol. 2021;20(10):795–820. 10.1016/S1474-4422(21)00252-0 PMC8443449 34487721

[2] Wen-Jun T, Zhenping Z, Peng Y, Lei C, Jingsheng Z, Huisheng C, et al. Estimated burden of stroke in China in 2020. JAMA Netw Open. 2023;6(3):e231455. 36862407 10.1001/jamanetworkopen.2023.1455 PMC9982699

[3] Rui G, Renjie Z, Ran L, Yi L, Hao L, Lu M, et al. Machine learning-based approaches for prediction of patients’ functional outcome and mortality after spontaneous intracerebral hemorrhage. J Pers Med. 2022;12(1):112. 35055424 10.3390/jpm12010112 PMC8778760

[4] Koutarou M, Kazuaki I, Katsuhiko M, Koki T, Shigeo Y, Hidehisa S, et al. Machine learning-based prediction for in-hospital mortality after acute intracerebral hemorrhage using real-world clinical and image data. J Am Heart Assoc. 2024;13(24):e036447. 39655759 10.1161/JAHA.124.036447 PMC11935536

[5] Xianli Q, Yong H, Changchun C, Yuheng L, Haofei H. Association between atherogenicity indices and prediabetes: a 5-year retrospective cohort study in a general Chinese physical examination population. Cardiovasc Diabetol. 2025;24:220. 40399916 10.1186/s12933-025-02768-8 PMC12096774

[6] Ronda L, Vignan Y, Tim R, Michel S, Robert F, Magdy HS, et al. Predicting long-term outcomes in acute intracerebral haemorrhage using delayed prognostication scores. Stroke Vasc Neurol. 2021;6(4):536–41. 33758069 10.1136/svn-2020-000656 PMC8717768

[7] Han F, Dongjiang H, Ran X, Qian Y, Hang L, Qing Y, et al. Risk prediction models for deep venous thrombosis in patients with acute stroke: A systematic review and meta-analysis. Int J Nurs Stud. 2023;149:104623. 37944356 10.1016/j.ijnurstu.2023.104623

[8] Larissa S, David M, Mike C, Davina G, Alessandro L, Mark P, et al. Preferred reporting items for systematic review and meta-analysis protocols (PRISMA-P) 2015: elaboration and explanation. BMJ. 2015;350:g7647. 25555855 10.1136/bmj.g7647

[9] Miranda C, Tianjing L, Matthew J P, Jacqueline C, Vivian A W, Julian Pt H, et al. Updated guidance for trusted systematic reviews: a new edition of the Cochrane Handbook for Systematic Reviews of Interventions. Cochrane Database Syst Rev. 2019;10:ED000142. 10.1002/14651858.ED000142 PMC10284251 31643080

[10] Jawed N, Helge K, Sarah E, Constanze F, Peter S, Thilo R, et al. Imaging-based outcome prediction of acute intracerebral hemorrhage. Transl Stroke Res. 2021;12:958–67. 33547592 10.1007/s12975-021-00891-8 PMC8557152

[11] Carlos F-L, Pablo H, Virginia M-A, Manuel R-Y, Sonia S-G, Iria L-D, et al. Random forest-based prediction of stroke outcome. Sci Rep. 2021;11:10071. 33980906 10.1038/s41598-021-89434-7 PMC8115135

[12] Obaid Ur Rehman K, Hanzala Ahmed F, Rayyan N, Hamna H. Advancements in prognostic markers and predictive models for intracerebral hemorrhage: from serum biomarkers to artificial intelligence models. Neurosurg Rev. 2024;47:382. 10.1007/s10143-024-02635-2 39083096

[13] Weixiong Z, Jiaying C, Linling S, Genghong X, Jiahui X, Shuqiong Z, et al. Clinical, radiological, and radiomics feature-based explainable machine learning models for prediction of neurological deterioration and 90-day outcomes in mild intracerebral hemorrhage. BMC Med Imaging. 2025;2:184. 10.1186/s12880-025-01717-x PMC12105149 40420050

[14] Ying S, Yuying L, Xiening X. Impact of emergency fast track on treatment time and outcomes in acute stroke: a systematic review and meta-analysis. BMC Emerg Med. 2025;25:186. 40993515 10.1186/s12873-025-01336-3 PMC12462200

[15] Karel G M M, Johanna A A D, Tabea K, Lotty H, Constanza AN, Paula D, et al. PROBAST+AI: an updated quality, risk of bias, and applicability assessment tool for prediction models using regression or artificial intelligence methods. BMJ. 2025;388:e082505. 10.1136/bmj-2024-082505 PMC11931409 40127903

[16] Rachel YLK, Conrad H, Terry-Ann C, Benjamin J, Alexander F, David C, et al. Artificial Intelligence in Fracture Detection: A Systematic Review and Meta-Analysis. Radiology. 2022;304(1):50–62. 35348381 10.1148/radiol.211785 PMC9270679

[17] Caspar J VL, Sara vE, Eli-Boaz C. Selecting relevant moderators with Bayesian regularized meta-regression. Res Synth Methods. 2023;14(2):301–322. 10.1002/jrsm.1628 36797984

[18] Cheng-Chang Y, Oluwaseun Adebayo B, Lung C, Jia-Hung C, Chien-Tai H, Yi-Ting H, et al. Risk factor identification and prediction models for prolonged length of stay in hospital after acute ischemic stroke using artificial neural networks. Front Neurol. 2023;14:1085178. 10.3389/fneur.2023.1085178 PMC9947790 36846116

[19] Gordon HG, Andrew DO, Gunn EV, Regina K, Yngve F-Y, Pablo A-C, et al. GRADE: an emerging consensus on rating quality of evidence and strength of recommendations. BMJ. 2008;336(7650):924–6. 18436948 10.1136/bmj.39489.470347.AD PMC2335261

[20] Tânia M, Vera L, Dulce C, Eduardo S. The Effectiveness of Psychoeducational Interventions in Adolescents’ Anxiety: A Systematic Review Protocol. Nurs Rep. 2022;12(1):217–25. 35324568 10.3390/nursrep12010022 PMC8950651

[21] Koutarou M, Masahiro S, Kazuaki I, Koki T, Katsuhiko M, Jenhui C, et al. Performance of multimodal prediction models for intracerebral hemorrhage outcomes using real-world data. Int J Med Inform. 2025;202:105989. 40412140 10.1016/j.ijmedinf.2025.105989

[22] Kerry EP, Vincent MT, Lu L, Muhammad W, Armond J, Lee C, et al. Classification models using circulating neutrophil transcripts can detect unruptured intracranial aneurysm. J Transl Med. 2020;18:392. 33059716 10.1186/s12967-020-02550-2 PMC7565814

[23] Mohamed Sobhi J, Olivier J, David K, George H, Alejandro R, Thien H, et al. Interpretable machine learning modeling for ischemic stroke outcome prediction. Front Neurol. 2022;13:884693. 35665041 10.3389/fneur.2022.884693 PMC9160988

[24] Wenqing T, Li Z, Yueqi Z. Intracranial hemorrhage prediction in acute ischemic stroke patients with anterior circulation tandem lesions following endovascular thrombectomy. Front Neurol. 2025;16:1598203. 40948636 10.3389/fneur.2025.1598203 PMC12426950

[25] Amir Mahmoud A, Mohammad Amin A, Nima BL, Danial E, Benyamin G, Mahsa V, et al. Application of deep learning for predicting hematoma expansion in intracerebral hemorrhage using computed tomography scans: a systematic review and meta-analysis of diagnostic accuracy. Radiol Med. 2025;130(12):1973–85. 40932678 10.1007/s11547-025-02089-6

[26] Khalid S, Ahmed YA, Mostafa Hossam El Din M, Ibrahim S, Abdallah A, Ahmed ES. Automated Emergent Large Vessel Occlusion Detection Using Viz.ai Software and Its Impact on Stroke Workflow Metrics and Patient Outcomes in Stroke Centers: A Systematic Review and Meta-analysis. Transl Stroke Res. 2025(16):2258–227. 10.1007/s12975-025-01354-0 PMC12596299 40335883

[27] Adrian M, Dhaval K, Vaibhav A, Kristin S, Eduardo C, Christian lF, et al. Optimizing stroke lesion segmentation: A dual-approach using Gaussian mixture models and nnU-Net. Comput Biol Med. 2025;192:110221. 10.1016/j.compbiomed.2025.110221 40318493

[28] Chaonan D, Cong W, Zhiwei L, Wenxuan X, Qizhe Z, Alleyar A, et al. Machine learning algorithms integrate bulk and single-cell RNA data to unveil oxidative stress following intracerebral hemorrhage. Int Immunopharmacol. 2024;137:112449. 38865753 10.1016/j.intimp.2024.112449

[29] Sergei P. Advancing AI in healthcare: A comprehensive review of best practices. Clin Chim Acta. 2023;548:117519. 37595864 10.1016/j.cca.2023.117519

[30] Marco C, Dor A, Huanwen C, Keren Z, Michal G, Christopher JL, et al. Estimation of Ventricular and Intracranial Hemorrhage Volumes and Midline Shift on an External Validation Data Set Using a Convolutional Neural Network Algorithm. Neurosurgery. 2025;97(3):719–26. 40227036 10.1227/neu.0000000000003455

[31] Alexandra LC, Sunil AS. Overview of Imaging Modalities in Stroke. Neurology. 2021;97(20 Suppl 2):S42–51. 10.1212/WNL.0000000000012794 PMC11418094 34785603

[32] Gary SC, Johannes BR, Douglas GA, Karel GMM. Transparent Reporting of a multivariable prediction model for Individual Prognosis or Diagnosis (TRIPOD): the TRIPOD statement. Ann Intern Med. 2015;162(1):55–63. 25560714 10.7326/M14-0697

[33] Gary SC, Paula D, Constanza LAN, Jie M, Lotty H, Johannes BR, et al. Protocol for development of a reporting guideline (TRIPOD-AI) and risk of bias tool (PROBAST-AI) for diagnostic and prognostic prediction model studies based on artificial intelligence. BMJ Open. 2021;11(7):e048008. 10.1136/bmjopen-2020-048008 PMC8273461 34244270

