# Supplementary material for: Assessing performance, calibration, and explainability of machine learning versus traditional models for early outcome prediction after spontaneous intracerebral hemorrhage: a systematic review and meta-analysis protocol
Source: Syst Rev. 2026 Jan 10;15:48. doi: 10.1186/s13643-025-03059-9 (PMC12882189; doi:10.1186/s13643-025-03059-9)
Supplement: Supplementary file 1 — Supplementary Material 1. Search Strategy. Detailed database search strategies including MeSH/Emtree terms and Boolean operators. [file 13643_2025_3059_MOESM1_ESM.pdf]

Database	Step No	Search Terms
PubMed	1	("Intracerebral Hemorrhage"[Mesh] OR "Brain Hemorrhage"[Mesh] OR "intracerebral haemorrhage"[tiab] OR "ICH"[tiab] OR "cerebral hemorrhage"[tiab] OR "spontaneous intracerebral hemorrhage"[tiab])
	2	("Machine Learning"[Mesh] OR "Artificial Intelligence"[Mesh] OR "deep learning"[tiab] OR "neural network*"[tiab] OR "random forest"[tiab] OR "support vector machine"[tiab] OR "gradient boosting"[tiab] OR "predictive model*"[tiab] OR "risk prediction"[tiab])
	3	("Prognosis"[Mesh] OR "Treatment Outcome"[Mesh] OR "Outcome Assessment (Health Care)"[Mesh] OR "mortality"[tiab] OR "functional outcome"[tiab] OR "poor outcome"[tiab] OR "early outcome"[tiab])
	4	("logistic regression"[tiab] OR "Cox regression"[tiab] OR "clinical score"[tiab] OR "traditional model"[tiab] OR "statistical model"[tiab])
	5	#1 AND #2 AND #3
	6	#1 AND #2 AND #3 AND #4
Embase	1	('intracerebral hemorrhage'/exp OR 'brain hemorrhage'/exp OR 'spontaneous intracerebral hemorrhage':ti,ab,kw OR 'ICH':ti,ab,kw)
	2	('machine learning'/exp OR 'artificial intelligence'/exp OR 'deep learning':ti,ab,kw OR 'neural network*':ti,ab,kw OR 'random forest':ti,ab,kw OR 'support vector machine':ti,ab,kw OR 'predictive model*':ti,ab,kw OR 'risk prediction':ti,ab,kw)
	3	('prognosis'/exp OR 'treatment outcome'/exp OR 'mortality':ti,ab,kw OR 'functional outcome':ti,ab,kw OR 'early outcome':ti,ab,kw)
	4	('logistic regression':ti,ab,kw OR 'Cox regression':ti,ab,kw OR 'clinical score':ti,ab,kw OR 'traditional model':ti,ab,kw)
	5	#1 AND #2 AND #3 AND #4 NOT ('animal'/exp NOT 'human'/exp)
Cochrane CENTRAL	1	[mh "Intracerebral Hemorrhage"] OR ("spontaneous intracerebral hemorrhage":ti,ab,kw OR "ICH":ti,ab,kw)
	2	[mh "Machine Learning"] OR [mh "Artificial Intelligence"] OR ("deep learning":ti,ab,kw OR "neural network*":ti,ab,kw OR "predictive model*":ti,ab,kw)
	3	[mh "Prognosis"] OR ("functional outcome":ti,ab,kw OR "mortality":ti,ab,kw OR "early outcome":ti,ab,kw)
	4	(#1 AND #2 AND #3)
Scopus	1	TITLE-ABS-KEY("intracerebral hemorrhage" OR "ICH" OR "brain hemorrhage") AND TITLE-ABS-KEY("machine learning" OR "artificial intelligence" OR "deep learning" OR "predictive model*" OR "risk prediction") AND TITLE-ABS-KEY("prognosis" OR "mortality" OR "functional outcome" OR "early outcome") AND TITLE-ABS-KEY("logistic regression" OR "Cox regression" OR

		"traditional model")
	2	(LIMIT-TO(DOCTYPE, "ar") OR LIMIT-TO(DOCTYPE, "re")) AND (LIMIT-TO(LANGUAGE, "English"))
Web of Science	1	TS=("intracerebral hemorrhage" OR "ICH" OR "brain hemorrhage") AND TS=("machine learning" OR "artificial intelligence" OR "deep learning" OR "predictive model*" OR "risk prediction") AND TS=("prognosis" OR "mortality" OR "functional outcome" OR "early outcome") AND TS=("logistic regression" OR "traditional model" OR "clinical score")
IEEE Xplore	1	("intracerebral hemorrhage" OR "ICH" OR "brain hemorrhage") AND ("machine learning" OR "deep learning" OR "artificial intelligence") AND ("prediction" OR "prognosis" OR "outcome")
CNKI (中国知网)	1	TKA%=('自发性脑出血' + '脑出血' + '颅内出血') AND TKA%=('机器学习' + '深度学习' + '人工智能' + '预测模型' + '风险预测') AND TKA%=('预后' + '结局' + '功能恢复' + '病死率') AND TKA%=('逻辑回归' + '传统模型' + '统计模型') NOT TKA%=('动物实验' + '综述' + 'Meta 分析')
Wanfang (万方)	1	主题:(自发性脑出血 OR 脑出血 OR 颅内出血) AND 主题:(机器学习 OR 深度学习 OR 人工智能 OR 预测模型 OR 风险预测) AND 主题:(预后 OR 结局 OR 死亡率 OR 功能预后) AND 主题:(逻辑回归 OR 传统模型 OR 统计模型) NOT 主题:(动物 OR 综述 OR Meta 分析)
VIP (维普)	1	篇关摘：(自发性脑出血 OR 脑出血 OR 颅内出血)
	2	篇关摘：(机器学习 OR 深度学习 OR 人工智能 OR 预测模型 OR 风险预测)
	3	篇关摘：(预后 OR 功能结局 OR 死亡率)
	4	篇关摘：(逻辑回归 OR 传统模型 OR 统计学模型)
	5	(#1 AND #2 AND #3 AND #4) NOT 篇关摘：(动物 OR 综述 OR Meta 分析)
CBM (中国生物医学文献数据库)	1	('自发性脑出血'[不加权:扩展] OR '脑出血'[不加权:扩展] OR '颅内出血'[不加权:扩展]) AND ('机器学习'[不加权:扩展] OR '深度学习'[不加权:扩展] OR '人工智能'[不加权:扩展] OR '预测模型'[不加权:扩展]) AND ('预后'[不加权:扩展] OR '结局'[不加权:扩展] OR '死亡率'[不加权:扩展]) AND ('逻辑回归'[不加权:扩展] OR '传统模型'[不加权:扩展]) NOT ('动物'[不加权:扩展] OR '综述'[文献类型] OR 'Meta 分析'[文献类型])
